# *Clonostachys rosea* demethiolase STR3 controls the conversion of methionine into methanethiol

**DOI:** 10.1038/srep21920

**Published:** 2016-02-23

**Authors:** Kai-Zhi Jia, Quan Zhang, Lin-Yang Sun, Yang-Hua Xu, Hong-Mei Li, Ya-Jie Tang

**Affiliations:** 1 Key Laboratory of Fermentation Engineering (Ministry of Education), Hubei Provincial Key Laboratory of Industrial Microbiology, Hubei Provincial Cooperative Innovation Center of Industrial Fermentation, Hubei University of Technology, Wuhan 430068 China

## Abstract

Eukaryote-derived methioninase, catalyzing the one-step degradation of methionine (Met) to methanethiol (MTL), has received much attention for its low immunogenic potential and use as a therapeutic agent against Met-dependent tumors. Although biological and chemical degradation pathways for Met-MTL conversion are proposed, the concrete molecular mechanism for Met-MTL conversion in eukaryotes is still unclear. Previous studies demonstrated that α-keto-methylthiobutyric acid (KMBA), the intermediate for Met-MTL conversion, was located extracellularly and the demethiolase STR3 possessed no activities towards Met, which rule out the possibility of intracellular Met-MTL conversion pathway inside eukaryotes. We report here that degradation of Met resulted in intracellular accumulation of KMBA in *Clonostachys rosea*. Addition of Met to culture media led to the production of MTL and downregulation of *STR3,* while incubation of Met with surrogate substrate α-ketoglutaric acid enhanced the synthesis of MTL and triggered the upregulation of *STR3*. Subsequent biochemical analysis with recombinant STR3 showed that STR3 directly converted both Met and its transamination product KMBA to MTL. These results indicated that STR3 as rate-limiting enzyme degrades Met and KMBA into MTL. Our findings suggest STR3 is a potential target for therapeutic agents against Met-dependent tumors and aging.

Methionine (Met) is employed at multiple levels in cellular metabolism: as a protein constituent, in the initiation of mRNA translation, and as a regulatory molecule in the form of *S*-adenosylmethionine. Thus, it can be assumed that Met synthesis, accumulation, and transfer play fundamental roles in cell growth and development^1^,^2^,^3^,^4^. Despite the importance of Met, Met catabolism is not fully understood especially in eukaryotic cells, most likely because of the undetected transient intermediates and functional redundancy of enzymes within the pathway^5^,^6^.

In eukaryotic cells, Met was catabolized to α-keto-methylthiobutyric acid (KMBA) by aminotransferase, KMBA was hypothesized to be converted to methional and methionol via Ehrlich pathway and methanethiol (MTL) via demethiolation pathway, respectively^7^. However, other studies suggested that KMBA accumulated only extracellularly, proposing that KMBA was highly toxic to cells and actively excreted^6^,^7^,^8^. It is thus currently believed that the degradation of KMBA into volatile organic sulfur-containing compounds (VOSCs) including methional, methanol, MTL and so on is an exclusively chemical reaction dependent on the physicochemical properties of the extracellular medium including pH, redox status, and ionic strength^6^.

Met degradation pathway genes are highly conserved, Ehrlich pathway genes including aminotransferase genes *AAT1*, *AAT2*, *ARO8*, *ARO9, BAT1, BAT2*, and α-ketoacid decarboxylase gene *PDC* are annotated in fungi based on the homology to *Saccharomyces cerevisiae* genes^6^,^8^,^9^,^10^. Demethiolation pathway enzymes CYS3 and STR3, annotated as cystathionine β-lyase and γ-lyase, are inferred as the key enzymes directly converting KMBA to MTL for both compounds contain C-S bond^11^,^12^,^13^. While *CYS3* only exited in the genome of *S. cerevisiae*, its homologue in *C. rosea* were not obtained after searching *C. rosea* genome^13^. Recombinant *S. cerevisiae* CYS3 and STR3 expressed and purified from *E. coli* displayed a broad specificity toward cysteine-S-conjugates except Met^13^,^14^. To date, no gene has been identified for the conversion of Met and its transamination product KMBA into MTL.

Methioninases, catalyzing the one-step degradation of Met to MTL, α-ketobutyrate (α-KB) and ammonium, have received much attention as therapeutic agents against various Met-dependent tumors and aging^15^,^16^,^17^,^18^,^19^,^20^. The therapeutic role is attributed to reducing Met concentration in media deprived tumor cell of dependence on external Met, and trigger an “amino acid response” to allow normal cells to adapt to their environment^16^,^17^,^19^,^21^. More importantly, methioninase transfected into cancer cells evinced either moderate cell death or severe aggregation^18^. However, these methioninases are of bacterial origin, and usually associated with high immunogenicity, low substrate specificity, and hazardous effects on the kidney and liver^17^,^22^,^23^,^24^. The proposed methioninase in fungi needs to be functionally defined for its potential to overcome these defects.

In this study, we located the biodegradation pathway of Met, and then used genetic, biochemical and metabolite–based analysis to prove that STR3 has the ability for one-step degradation of Met and its transamination product KMBA in *Clonostachys rosea*. Our findings support an effective approach against various Met-dependent tumors and aging.

## Results

### KMBA, the intermediate for Met-MTL conversion, is located intracellularly

**Figure 1:**
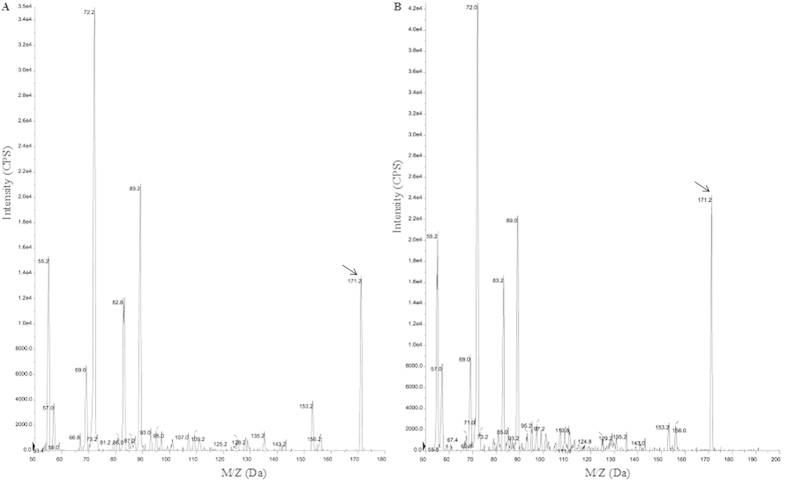
Intracellular KMBA was identified by LC-MS. (**A**) LC-MS analysis of standard KMBA, (**B**) LC-MS analysis of intracellular KMBA.

**Figure 2:**
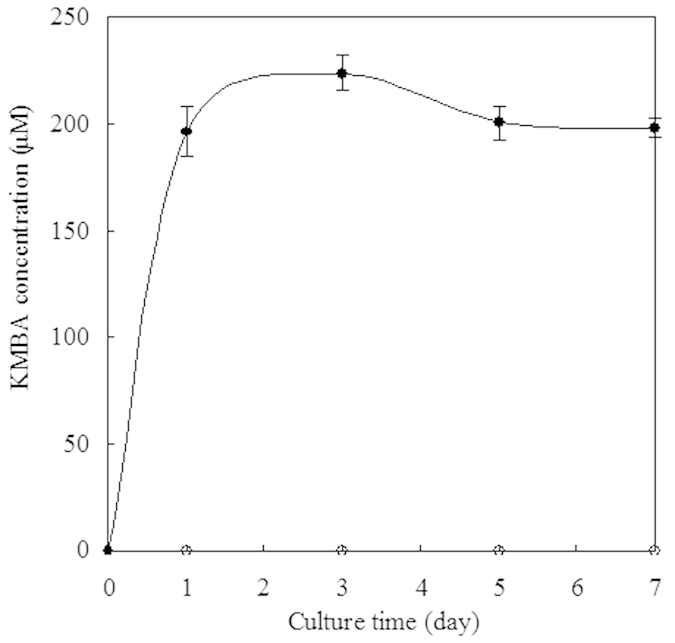
KMBA was located exclusively intracellularly in *C. rosea*. (●intracellular KMBA, ○extracellular KMBA).

A hypothesis of KMBA-MTL conversion by physicochemical or enzymatic degradation of Met transamination product KMBA was proposed^6^,^7^,^8^. The extracellular or intracellular location of KMBA is a key indicator of KMBA-MTL conversion^6^. Therefore, we investigated 30 fungal strains in our laboratory to detect KMBA. The possible intracellular KMBA was only found in *C. rosea* Tang19, liquid chromatography and LTQ-Orbitrap MS analyses demonstrated that the metabolite showed similar molecular weight as KMBA (Fig. 1A,B). Subsequently, intracellular and extracellular KMBA was detected by high-performance liquid chromatography for a period of 7 days. After 1 day, Met degradation resulted in the intracellular accumulation of KMBA. With longer incubation time, KMBA was found to be accumulated intracellularly but was undetectable in extracellular samples (Fig. 2). MTL and its derivatives were also detected during fermentation. Additionally, we did not observe the production of MTL from KMBA after 200 μM KMBA was added into the fermentation broth with mycelia removed (data not shown). These results indicate that KMBA is the intermediate of Met degradation inside cells, suggesting that intracellular Met-MTL conversion pathway existed in *C. rosea*.

### ARO8-2, BAT and STR3 are putative enzymes for Met-MTL conversion

**Figure 3:**
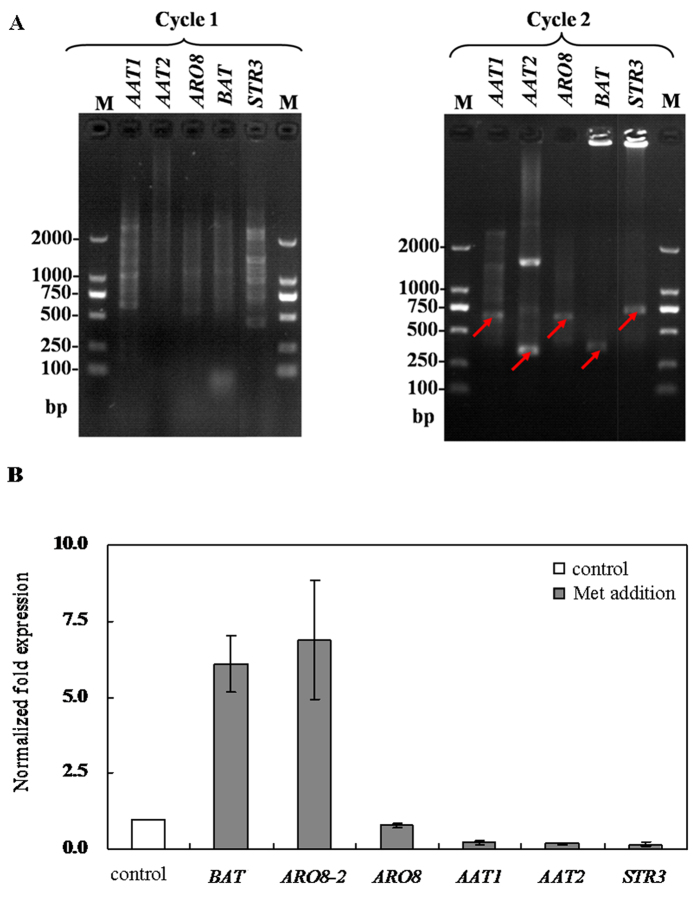
Role of *ARO8-2, BAT* and *STR3* in Met-MTL conversion. (**A**) Met-MTL conversion pathway genes obtained by homology-based cloning, (**B**) Transcriptional analysis of putative pathway genes (Normalized fold expression values for synthase genes were relative to the control without Met addition. The error type was standard deviation).

Aminotransferase and demethiolase are involved in Met-MTL conversion in *Ascomycetes* especially *Saccharomycetes*. To explore the intracellular conversion pathway in *C. rosea,* we mined Kyoto Encyclopedia of Genes and Genomes (KEGG) to retrieve the sets of aminotransferase and demethiolase, and conducted multiple alignments of amino acid sequences by ClustalX. Based on the analysis of the highly conserved regions, degenerate PCR primers were designed for cloning Met-MTL pathway gene homologs in *C. rosea* (Table 1). After two rounds of degenerate PCR, we found three groups of orthologous genes encoding aminotransferase, including aspartate amino acid aminotransferase genes *AAT1* and *AAT2*, aromatic acid aminotransferase genes *ARO8* and *ARO8-2*, and branched-chain amino acid aminotransferase genes *BAT* (Fig. 3A). By using NCBI blastX, the coding DNA sequences were aligned and the analysis results showed that *AAT1*, *AAT2*, *ARO8*, *ARO8-2* and *BAT* were 71%, 81%, 88%, 77% and 78% identical to the previously reported homologous genes of *Sordariomycetes* at amino acid level. Another gene ortholog encoding demethiolase was also successfully amplified, which showed 87% identity to *STR3* at the amino acid level (Fig. 3A). Subsequently, full-length cDNAs containing these Met-MTL pathway genes were isolated from cDNA library by RACE (rapid-amplification of cDNA ends) technology.

To better understand the role of these putative genes involved in Met-MTL conversion in *C. rosea*, we assessed the changes in gene transcriptional expression patterns after addition of 5 g/L Met into the culture media. The addition of Met resulted in upregulation of aminotransferase genes *BAT* and *ARO8-2* by 6.09 and 6.89 folds, while downregulation of aspartate amino acid aminotransferase genes *AAT1* and *AAT2*, and demethiolase gene *STR3* by 4.55, 5.88 and 6.25 folds, respectively (Fig. 3B). No obvious change was found in aromatic acid aminotransferase gene *ARO8* (Fig. 3B). These results were consistent with the observations in *Yarrowia lipolytica* and *Kluyveromyces lactis* that MTL biosynthesis was initiated by the upregulation of branched-chain amino acid aminotransferase genes and aromatic acid aminotransferase genes^9^,^25^. However, in contrast to the studies showing the effects of Met addition on transcriptional expression of *STR3* in *Kluyveromyces lactis* and *Yarrowia lipolytica*^8^,^9^, we found that MTL production induced by Met addition was accompanied with reduction of *STR3*, suggesting that STR3 may be a rate-limiting enzyme controlling Met-MTL conversion in *C. rosea*.

### STR3 is inferred as rate-limiting enzyme controlling Met-MTL conversion

**Figure 4:**
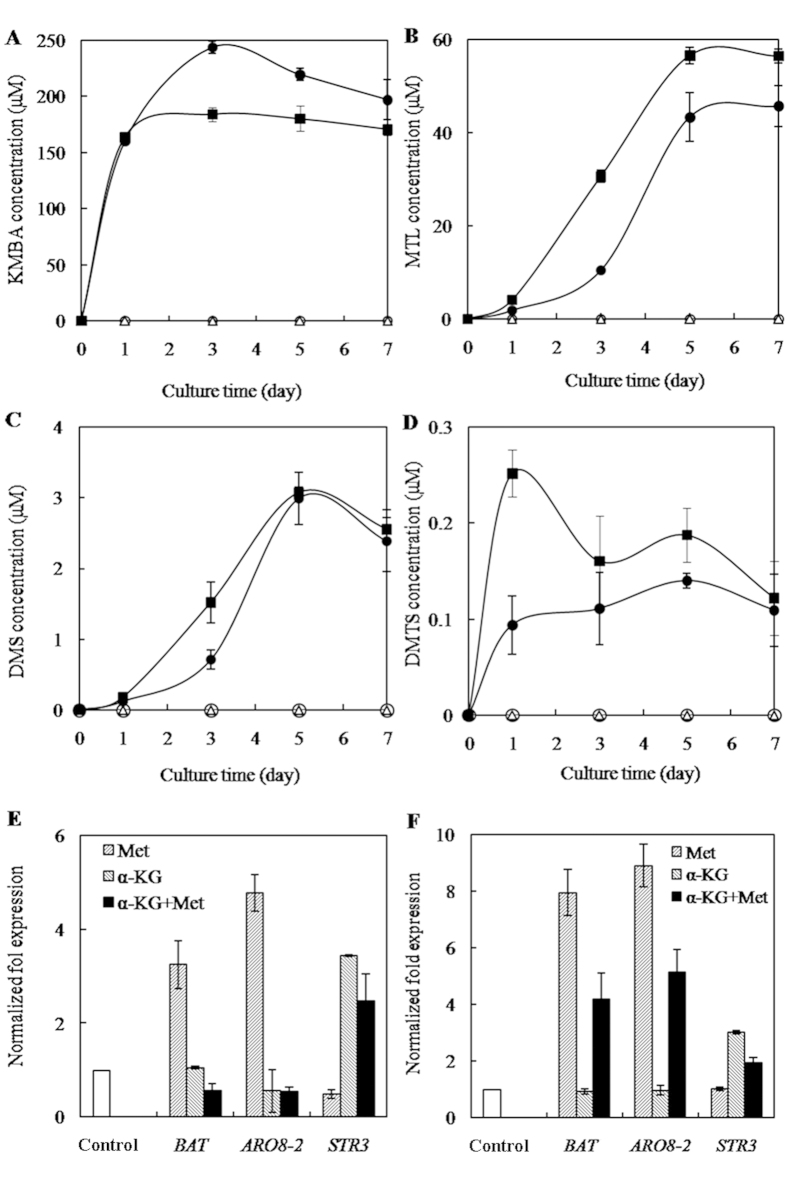
Functional analyses of *STR3* in Met-MTL conversion. (**A–D**) Effect of α-KG addition on the production of KMBA (**A**), MTL (**B**), DMS (**C**) and DMTS (**D**) (■The addition of α-KG with Met, ●The addition of Met, □The addition of α-KG, ○Control), (**E,F**) Effect of α-KG addition on transcriptional expression of Met-MTL conversion pathway genes on days 3 (**E**) and 5 (**F**) (Normalized fold expression values for synthase genes were relative to the control without Met addition. The error type was standard deviation).

MTL production was closely positively correlated with the transcriptional expression of Met-MTL pathway genes^6^,^7^,^8^,^9^,^26^. In addition, a previous study showed that addition of surrogate substrate α-ketoglutaric acid (α-KG) stimulated MTL production^28^. To further identify synthase genes involved in Met-MTL conversion in *C. rosea*, we added α-KG into fermentation media and analyzed the mRNA expression changes of possible pathway genes. The addition of α-KG alone induced the upregulation of *STR3*, but MTL and its derivatives were not detected in the fermentation media, which is due to the limited intracellular Met. Relative to L-Met addition alone, the incubation of L-Met with α-KG for 3 day reduced the production of KMBA (Met aminotransferation product) by 24.46%, and the mRNA levels of aminotransferase genes *BAT* and *ARO8-2* by 5.80 and 8.85 folds (Fig. 4A,E). However, the production of MTL and its derivatives DMS and DMTS was increased by 193.58%, 114.08% and 45.45%, and the demethiolase gene *STR3* was correspondingly upregulated by 5.06 fold (Fig. 4B–E). Similar effects of α-KG addition on the transcriptional expression of *BAT*, *ARO8-2* and *STR3*, and MTL production were observed after 5 days of incubation (Fig. 4F). These results demonstrated that the addition of α-KG inhibited the transaminase activity but stimulated the demethiolase activity. Correlation of the upregulation of *STR3* with the increased production of MTL and its derivatives even with the downregulation of *BAT* and *ARO8-2* indicated that *STR3* may be the key enzyme controlling Met-MTL conversion.

### STR3 catalyzes direct conversion of both Met and KMBA into MTL

**Figure 5:**
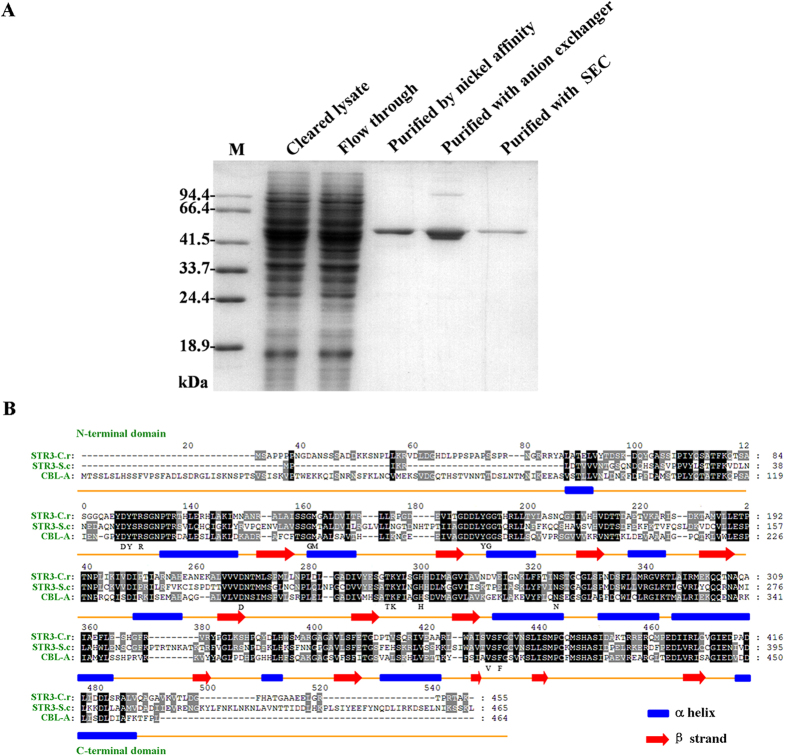
Functional analyses of STR3 *in vitro*. (**A**) SDS-PAGE of purified STR3, SEC: size exclusion chromatography; (**B**) Partial sequence alignment of STR3 with other demethiolases. Number is provided for aCBL, the catalytic residues (Y181, D253, K278), binding sites related to PLP (Y127, R129, G157, M158, T277), substrate binding sites (D126, G182, N307, V404, F406) in aCBL are indicated, STR3-C.r: *C. rosea* STR3(KT157524), STR3-S.c: *S. cerevisiae* STR3 (EDZ72299), CBL-A: Arabidopsis CBL (NP_191264).

Recombinant STR3 was expressed in *S. cerevisiae* and purified using Ni-nitrilotriacetic acid chromatography to capture the C-terminal six-histidine-tagged protein^27^. Subsequently, nearly pure STR3 was obtained by anion-exchange column and size-exclusion chromatography (Fig. 5A). Monomeric recombinant STR3 has a predicted molecular weight of 49 kDa, as determined by migration on an SDS-PAGE gel (Fig. 5A). The identity of the purified protein as the *STR3*-encoded protein was confirmed by MALDI-TOF MS (E value of 2.3e^−43^, sequence coverage 56%).

To further support our conclusion that STR3 is a rate-limiting enzyme controlling Met-MTL conversion, Met and its transamination product KMBA were used as substrates for STR3 in biochemical reactions. Except the controls, MTL was detected in reaction systems either with Met or KMBA, indicating that STR3 has the ability to convert Met and KMBA to MTL. STR3 had a Km of 196.37 mM for Met (Vmax = 0.034 μM·min^−1^) and 16.68 mM for KMBA (Vmax = 1.56 μM min^−1^), showing that STR3 can degrade Met and KMBA, but preferred the substrate KMBA to its precursor Met. This suggests that one-step degradation of Met into MTL exists, but the conversion of KMBA to MTL is the primary pathway in *C. rosea*.

STR3 protein contains 455 amino acids with 49% and 51% identity to Arabidopsis CBL (cystathionine β-lyase) and *S. cerevisiae* STR3, respectively. Both CBL and STR3 are PLP-dependent and selectively cleave the covalent bond at β- or γ-position of sulfur-containing amino acid substrates^29^. Based on Arabidopsis CBL structure^30^, STR3 is predicted to have an N-terminal PLP binding domain and similar binding or catalytic site residues as CBL (Fig. 5B).

### PLP dependence and cystathionine specificity

To validate that PLP is a cofactor in STR3 activity, different molar ratios between PLP and STR3 in enzymatic reactions were investigated to determine the demethiolation activity of STR3. Demethiolation activity was detected even without exogenous PLP addition, but the addition of exogenous PLP significantly enhanced STR3 activity (Table 2), which indicated that purified STR3 may carry intracellular PLP. This result was confirmed by detecting the characteristic absorbance of PLP at 420 nm^31^.

The other sulfur-containing amino acids with C_β, γ_-S bonds were further assayed as potential substrates for the STR3 enzyme (Table 3). Thionitrobenzoic acid derivatives produced by the reaction between thiol and 5, 5′-dithiobis-2-nitrobenzoic acid were detected in all reactions, suggesting that STR3 has β, γ-lyase activity with a broad range of substrate specificity. Furthermore, in all tested sulfur-containing amino acids, L-cystathionine was the most effective physiological substrate for STR3 (Table 3). It indicated that STR3 catalyzed the conversion of cystathionine to homocysteine, the precursor of Met.

## Discussion

**Figure 6:**
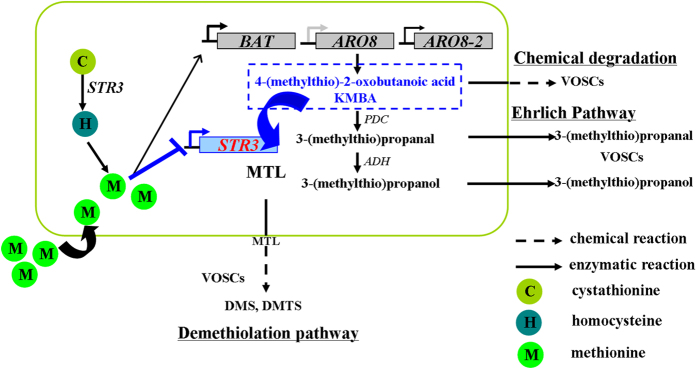
The Met-MTL conversion pathway proposed in the present study. The blue marks indicate proposed functions of STR3 in the present study. After Met addition, KMBA as a Met degradation intermediate was localized intracellularly; *BAT* and *ARO8-2* are activated, but *STR3* was repressed accompanied with MTL production; STR3 catalyzes direct conversion of Met and its transaminated product KMBA into MTL. VOSCs: volatile organic sulfur-containing compounds including 3-(methylthio)propanal, 3-(methylthio)propanol and MTL, DMS, DMTS.

In this study, we demonstrated that KMBA, the intermediate of Met-MTL conversion, was located intracellularly and STR3 is the key enzyme controlling Met-MTL conversion in *C. rosea.* Importantly, biochemical analysis with purified recombinant STR3 protein showed that STR3 catalyzed the direct conversion of both Met and KMBA to MTL (Fig. 6). Our findings indicate that STR3 is the key demethiolase controlling Met-MTL conversion in *C. rosea*.

The MTL branch of Met degradation pathways represents the basic metabolism in eukaryotes based on gene annotation studies^11^,^26^,^32^. Previous studies demonstrated that KMBA was located extracellularly and STR3 possessed no activities towards Met, which rule out the possibility of intracellular Met-MTL conversion pathway inside eukaryotes^6^,^8^,^14^,^26^. Therefore, the precise Met-MTL conversion pathway remains unclear. Our genetic, biochemical and metabolic studies demonstrated that KMBA is located intracellularly and STR3 is a key demethiolase in Met-MTL conversion in *Clonostachys rosea*, unraveling the complex metabolic pathway of Met in eukaryotes.

Aminotransferases involved in the conversion of Met into MTL are highly conserved in fungi^5^,^6^,^8^,^10^,^26^. Upregulation of branched-chain amino acid aminotransferase gene *BAT1* and aromatic acid aminotransferase gene ARO8 led to higher MTL production^6^,^8^,^9^,^27^,^33^. However, the functional role of demethiolase STR3 in the process of MTL biosynthesis is still controversial as different studies reported contrasting results when fermentation strains were inoculated into different meida^9^,^14^,^33^. In this study, addition of Met triggered the downregulation of demethiolase gene *STR3*, which is consistent with the result in *Yarrowia lipolytica*^9^,^33^. In contrast, the addition of Met together with α-KG reversed the effect of Met alone, and the elevated *STR3* expression was accompanied with increased MTL production, suggesting that STR3 is a rate-limiting enzyme controlling the conversion of Met into MTL.

Biochemical analyses indicated that STR3 exhibited the ability for one-step degradation of Met and its transamination product KMBA, revealing a novel enzymatic role distinct from its previously identified features as a eukaryotic family member. Met restriction suppressed cancer cell metabolism and extended lifespan of normal cell^15^,^19^,^20^,^21^. Due to the low immunogenicity of *C. rosea* STR3, enzymatic or dietary depletion of Met *in vivo* makes STR3 an ideal therapeutic candidate against various types of Met-dependent tumors and aging^17^,^18^,^34^,^35^,^36^. These PLP-dependent enzymes share an appreciable degree of sequence homology and exhibit similarities in their three-dimensional structures^37^,^38^. Amino acid residues at the enzyme active site rather than the structural fold control substrate specificity and reaction type^29^,^39^. *C. rosea* STR3 has similar amino acid residues at the enzyme active site, substrate binding site and cofactor binding site as Arabidopsis CBL. Further analysis of the different amino acid sites of STR3 is required to understand the substrate specificity for Met. Additionally, STR3 is a cystathionine β-lyase (CBL) with a higher catalytic efficiency for L-cystathionine than other physiological substrates. In this catalytic reaction, cystathionine is cleaved by STR3 to produce homo-Cys, the direct precursor of Met in eukaryotes^2^,^30^. *STR3* expression repressed by Met addition may be related with feedback effects. Thus, STR3 is a potential key target for the biosynthesis of Met and MTL in eukaryotes by metabolic engineering^40^,^41^.

In conclusion, STR3 displayed demethiolase activities to Met and its transamination product KMBA. Together with genetic and metabolite data, we demonstrated that the direct conversion of Met and its transamination product KMBA into MTL controlled by STR3 occurs in *C. rosea*. Our findings provide a rationale for Met depletion *in vivo* or dietary supplementation of methioninase in cancer therapy and anti-aging, and the regulation of essential amino acid Met content and volatile flavor constituent MTL in plants and yeast, respectively.

## Methods

### Chemicals

Chemical standards of MTL and its derivatives DMS and DMTS with over 98% purity were purchased from J&K Scientific Ltd (Beijing, China). All analytical grade reagents for STR3 enzymatic assay were purchased from Biosharp (Shenzhen, China), Tokyo Chemical Industry (Shanghai, China) and Sigma-Aldrich (St. Louis, MO).

### Microbial strains, media, and culture conditions

Chemically competent *E. coli* DH5α cells (TransGen Biotech, Beijing, China) were used for amplification of pYES2 plasmid (Invitrogen). Growth and selection were carried out in Luria-Bertani medium supplemented with 100 mg/L ampicillin. Commercial *S. cerevisiae* strain INVSc1 (Invitrogen) was used as a host strain for the expression of *STR3*. Yeasts were cultivated at 30 °C in either a rich medium YPD (Yeast Extract Peptone Dextrose medium, containing 1% (w/v) yeast extract, 2% peptone, and 2% glucose), or a synthetic minimal defined medium SC (synthetic complete medium, containing 2% glucose or raffinose, 0.67% yeast nitrogen base without amino acids (Biosharp)). Uracil (SC-U) was omitted to obtain selective plates for growing pYES2 transformants.

Thirty isolated fungal strains were grown on potato-agar-dextrose slants, precultured in fermentation medium (sucrose, 35 g/L; peptone, 2.5 g/L; yeast extract, 2.5 g/L; KH_2_PO_4_ ·H_2_O, 1 g/L; MgSO_4_·7H_2_O 0.5 g/L; Vitamin B1, 0.05 g/L), and then their ability to degrade Met was tested by inoculating them in fermentation medium added with 5 g/L Met. Met transamination product KMBA was only detected in the culture inoculated with *Clonostachys rosea* Tang 19, which was identified by ITS sequence (Genbank accession number: KT007105). This strain was selected for further study.

### Extraction and identification of KMBA by LC-MS

Intracellular KMBA was extracted as follows^6^. Fungal mycelia cultured in fermentation medium with 5 g/L Met were collected by filtration, washed with 20 mL of cold ultrapure water, and then filtered again. Mycelial pellets were stored at −80 °C. The pellets were resuspended with 1 mL of 1% (v/v) formic acid, incubated for 10 min at 95 °C and centrifuged for 30 min at 4 °C. The supernatant was lyophilized and stored at −80 °C. Before injection, samples were resuspended in water containing 0.1% formic acid and stored at 4 °C. Chromatographic separation was performed on a Reprosil-Pur Basic C18 column (4.6 mm × 250 mm × 5 μm) from Dr. Maisch GmbH (Germany) using a liquid chromatography 20AD system (Shimadzu Corporation, Japan). The optimized mobile phase was 0.1% formic acid water solution. The column oven temperature was set to 30 °C, and the flow rate was 1 mL/min. The detection wavelength was 197 nm. The retention time for KMBA was 12.5 min. For the determination of extracellular KMBA, 1 mL fermentation broth was centrifuged at 13,000 g for 30 min and then filtered through a 0.22 μm filter prior to detection.

Chromatographic purification of KMBA was carried on Amethyst C18-H column (10 mm × 250 mm × 5 μm) from Sepax Technologies (Newark, DE). Targeted samples were identified by comparison with the standard compounds, followed by tandem mass spectrometry (MS/MS) on a Finnigan TSQ Quantum Ultra AM instrument (Thermal, USA). The metabolites were confirmed by standard MS using positive electrospray ionization and scanned in the normal mass range from 50 m/z to 180 m/z. Characteristic fragment ions were detected by second-order MS.

### Cloning of Met-MTL pathway genes

The coding sequences of Met-MTL pathway genes including aminotransferase genes *AAT1*, *AAT2*, *BAT* and *ARO8*, and demethiolase gene *STR3* of *C. rosea* Tang 19 were amplified by PCR using degenerated forward/reverse primers (Table 1) designed on the basis of conserved regions of fungi in the previously reported sequences^5^,^6^,^9^. The full-length cDNAs encoding Met-MTL pathway genes were obtained by 5′ and 3′ RACE using nested primers (Table 1). The prediction of open reading frames (ORF) of these genes was conducted using the program ORF finder at the NCBI web site. Genbank numbers of *AAT1*, *AAT2*, *BAT*, *ARO8*, *ARO8-2* and *STR3* were KT157525, KT157520, KT157521, KT157522, KT157523, KT157524, respectively.

### Effect of Met and α-KG addition on the conversion of Met to MTL

The *C. rosea* culture was initiated by the addition of 0.5% (w/v) L-Met or 0.098% (w/v) α-KG (Sigma-Aldrich) with respective controls included. After culturing for 3 to 5 days in the fermentation medium, *C. rosea* mycelia were collected and the transcriptional expression of Met-MTL pathway genes was analyzed by quantitative real-time PCR. RNA was isolated using the MasterPure Fungal RNA Purification Kit (Biozym Scientific GmbH). RNA concentration and purity (A260/A280 ratio) were measured by using the Take3 micro-volume plate of BioTek Epoch Microplate Spectrophotometer. Complementary DNA (cDNA) was synthesized using a PrimeScriptTM 1^st^ Strand cDNA Synthesis Kit (TaKaRa Biotechnology (Dalian) Co., Ltd) with 0.5 μg of total RNA as the template. The 18S rRNA served as the reference gene for normalization of gene expression. Quantitative real-time PCR was performed on a StepOnePlus Real-Time PCR system in duplicate for at least three independent experiments. SYBR dye (TaKaRa Biotechnology (Dalian) Co., Ltd) was used to visualize gene amplification. The internal primers for RACE cloning were used to amplify the target sequence from different Met-MTL pathway genes (Table 1).

Met catabolic products including KMBA, MTL and its derivatives DMS and DMTS were determined after the cultures were incubated for 1, 3, 5 and 7 days. KMBA was extracted and detected following the above mentioned method. MTL and its derivatives were extracted and detected as follows. A 5-mL volume of fermentation culture was added into a 15-mL vial, the vial was tightly capped with a silicon septum and pre-equilibrated at 40 °C for 5 min, and then solid-phase microextraction (SPME) device was inserted into the headspace of vial to extracte MTL and its derivatives for 20 min at 40 °C. MTL and its derivatives were analyzed by gas chromatography (GC-2010 with a flame ionization detector (FID) from Shimadzu Technologies Inc., Tokyo, Japan) with an oven temperature program. First, the temperature was maintained at 35 °C for 3 min. Subsequently, the temperature reached 100 °C, with an increment of 8 °C/min. Then, the temperature was raised to 220 °C, with an increment of 10 °C for 1 min. Three independent cultures were analyzed for each condition.

### Expression and purification of STR3

The isolation and manipulation of DNA from *C. rosea* were performed using E.Z.N.A Fungal DNA kit (Omega) according to the manufacturer’s protocol. Primer pairs YSTR3-5H and YSTR3-3BH were designed to amplify the complete ORF of *STR3* from chromosomal DNA of *C. rosea* (Table 1). *Hind*III (in the forward primer) and *Bam*HI (in the reverse primer) restriction sites were introduced to ensure that *STR3* was correctly inserted into yeast expression plasmid pYES2. For purification of STR3, a His-tag encoding fragment was included before the stop codon (TTA). After the PCR product was cloned into pYES2, the resulting plasmid, pYES2-STR3, was transformed into *S. cerevisiae* strain INVSc1 as described^24^. Transformants were selected in SC medium lacking uracil (SC-U) and further confirmed by colony PCR with universal primers of F5 and R3.

After 20 h of galactose induction, yeast cultures were pelleted by centrifugation at 2,000 g for 5 min at 4 °C. Intracellular proteins were extracted by vigorously grinding the pelleted yeast cells cooled in liquid nitrogen. The lysates were centrifuged at 13,000 g for 1 h at 4 °C and filtered through a 0.22 μm (Millipore) filter. The resulting clear lysate was then exposed to nickel affinity chromatography using a 1-mL Ni-Sepharose 6 Fast Flow matrix according to the manufacturer’s instructions (GE Lifesciences). STR3 was purified by washing with 3 column volumes of NiA buffer containing 50 mM Tris, pH 8.5, 250 mM imidazole.

The collected sample was loaded on a Mono Q 10/100 GL (GE) equilibrated with buffer A (pH 7.5, 30 mM Tris-HCl, 15 mM NaCl, 1 mM EDTA, and 2 mM dithiothreitol). Only one peak was detected by using a linear gradient method, and was eluted with 516 mM NaCl. Subsequently, Size exclusion chromatography was carried out with a Superdex 200HR 10/30 analytical column (GE Lifesciences) using an AKTA^TM^ Pure 25 (FPLC, Pharmacia/GE Lifesciences). The flow rate was 0.4 mL/min. Proteins were separated by electrophoresis on sodium dodecyl sulfate-polyacrylamide gel electrophoresis (SDS-PAGE).

### Identification of STR3

STR3 resolved by SDS-PAGE was subjected to a trypsin digestion and analyzed by the 4700 Proteomics Analyzer MALDI-TOF/TOF (Applied Biosystems, Framingham, MA). Based on the combined MS and MS/MS spectra, STR3 was identified based on a 95% or higher confidence interval of their scores in the MASCOT V2.0 search engine (Matrix Science, London, U.K.). Functional roles of STR3 as demethiolase involved in the conversion of Met to MTL were measured using Met and its transamination product KMBA as substrates. Enzymatic reaction was conducted in a total reaction volume of 5 mL containing a final concentration of 1 mg/mL of STR3, 50 mM Tris-HCl (pH8.0), 5 μM PLP and 20 mM of Met or KMBA. Reaction mixtures were incubated for 1 h at pH 8.0. MTL production via demethiolation of Met and KMBA was determined by GC according to the method mentioned above. Control experiments referred to the complete assay with either omission of STR3 or with denatured STR3 (boiled for 10 min) and MTL and its derivatives were not detected after enzymatic reaction. Meanwhile, different concentrations of cofactor PLP (pyridoxal-5′-phosphate) were added to the standard enzyme assay mixture to investigate the effect of cofactor on STR3 activity toward KMBA. Demethiolating activities of STR3 were measured using the other sulfur-containing amino acids including S-methyl-L-cysteine, L-cystine, L-cystathionine and L-djenkolate as the substrates, as previously described^13^. After reactions were conducted in a total volume of 1 mL at pH 8.0 and 30 °C for 15 min, thionitrobenzoic acid derivatives produced by the reaction between thiol and 5, 5′-dithiobis-2-nitrobenzoic acid were measured spectrophotometrically at 412 nm. All the experiments were performed in triplicate.

## Additional Information

**How to cite this article**: Jia, K.-Z. *et al.* *Clonostachys rosea* demethiolase STR3 controls the conversion of methionine into methanethiol. *Sci. Rep.* **6**, 21920; doi: 10.1038/srep21920 (2016).

## Figures and Tables

**Table 1:** Primers for gene cloning and expression.

Genes	Primers	Sequence
Homology-based cloning		
* AAT1*	AAT1-5F	5′-ATHAAYYTNGGNGTNGGNGCNTA-3′
	AAT1-3R	5′-NARNCCNGTRTANGCRAACAT-3′
	AAT1-3R-22	5′-CCCATRTTYTTNGCRAANSWYTG-3′
* AAT2*	AAT2-5F	5′-YTNGGNATHGGNGCNTAGMGNGA-3′
	AAT2-3R	5′-DATNCKNCCNSWCATHGTNC-3′
	AAT2-5F-22	5′-CAYGCNTGYGCNCAYAAYCCNAC-3′
	AAT2-3R-22	5′-GGNGGRTTNSWDATYTCNSWNC-3′
* ARO8*	ARO8-5F2	5′-ATHWSNYTNGGNGGNGGNYTNCC-3′
	ARO8-3R-23	5′-RAACCANSWNCCNCKNGCNAC-3′
	ARO8-4R	5′-RTARTANGGYTCRTCYTCDATDAT-3′
* BAT*	BAT-5F1	5′-TAYGCNACNGARTGYTTYGARGG-3′
	BAT-3R1	5′-RTCNARNGGNGCNGTDATNA-3′
	BAT-5F2	5′-GNAARYTNMGNYTNTTYMGNCC-3′
	BAT-3R2	5′-RTARTTNGCNCCNACYTT-3′
* STR3*	STR3-5F	5′-MGNWSNGGNAAYCCNACNMG
	STR3-3R	5′-ARNSWRTTNACRCANCCRAA-3′
	STR3-3R2	5′-NCCNGTYTCRAANSWNARNACNGC-3′
Full-length cDNA cloning		
* AAT1*	AAT1-3RA1	5′-CCCTCGACAGGCTCGCCATTACCCAG-3′
	AAT1-3NRA2	5′-GTGCTGAGTTCCTTCAGCGCTGGTACAG-3′
	AAT1-5RA1	5′-CACTGGTCCTGGCTGGGGTCAACA-3′
	AAT1-5NRA2	5′-GAGGAGTGTTCTTGAGGTCCTCAACCAG-3′
* AAT2*	AAT2-3RA1	5′-GAGCTGCTCATCGCCCAGAGCTTC-3′
	AAT2-3NRA2	5′-GTGCTGGCTGCTTCCACGCCATCACCTC-3′
	AAT2-5RA1	5′-CTCACTCATTAGGCACCCCAGGC-3′
	AAT2-5NRA2	5′-CACAGGAAACAGCTATGACCATGATTAC-3′
* ARO8*	ARO8-3RA1	5′-TGACCTCTCCATCGCCCTCAACTAC-3′
	ARO8-3NRA2	5′-GAACACACCGAGCTGGTCTGCCGCCCTC-3′
	ARO8-5RA1	5′-TCGTCCATTGCCTCGGGGATGAGTC-3′
	ARO8-5NRA2	5′-GAACATGTTGATTCCAAGAGGGGCGAATG-3′
* ARO8-2*	ARO8-23RA1	5′-AGCTCCATGCAGCAGCGAGACCTTC-3′
	ARO8-23NRA2	5′-CCAGCCGGACTGGCTCATCCCTCAAGGGTG-3′
	ARO8-25RA1	5′-AGCGCAGAAGCTGGGCTGAGCCAC-3′
	ARO8-25NRA2	5′-CACTCTGGCCGTCTCGGAGATCGTGCTTG-3′
* BAT*	BAT-3RA1	5′-GAGCAGCGCGGATACTCCCTCTAC-3′
	BAT-3NRA2	5′-CCAGGCTCAGCCCTCCTCTATGTTATTG-3′
	BAT-5RA1	5′-GTGACGTACTGCTCCTCGCCGAAG-3′
	BAT-5NRA2	5′-CAGGTTCTGCTGGTGGCCCCTGGAGCTG-3′
* STR3*	STR3-3RA1	5′-TCCTCGAAACACCTACAAACCCTC-3′
	STR3-3NRA2	5′-CCAACAATCGCCCGGAATGCCCACGAAG-3′
	STR3-5RA1	5′-GAATGGCAAGAGTCTTGACTCCTC-3′
	STR3-5NRA2	5′-TCATTGGGTGACAGACCGCAGCCAGTTG-3′
*STR3* expression in *S. cerevisiae*		
* STR3*	YSTR3-5H	5′-CCCAAGCTTAACACAATGTCTGCCCCGCCTCCGCCAAATG-3′
	YSTR3-3BH	5′-CGCGGATCCTTAGTGGTGGTGGTGGTGGTGTTTGGCTGTGCGTGGAGTTCGTC-3′
	F5	5′-AGTATCAACAAAAAATTGTTA-3′
	R3	5′-GAATGTAAGCGTGACATAACTAA-3′

The restriction enzyme sites are underlined, and the His-tag encoding sequences are in bold.

**Table 2:** Effect of cofactor PLP levels on STR3 activity.

Molar ratio (PLP/STR3)	Relative activity (%)
0	100
0.5	132.6 ± 1.7
1	137.4 ± 2.5
2	140.7 ± 2.8

Specific activity toward KMBA was 0.53 ± 0.0030 μM MTL·h^−1^·mg protein^−1^. The relative activity of demethiolation of KMBA to MTL was set at 100%.

**Table 3:**
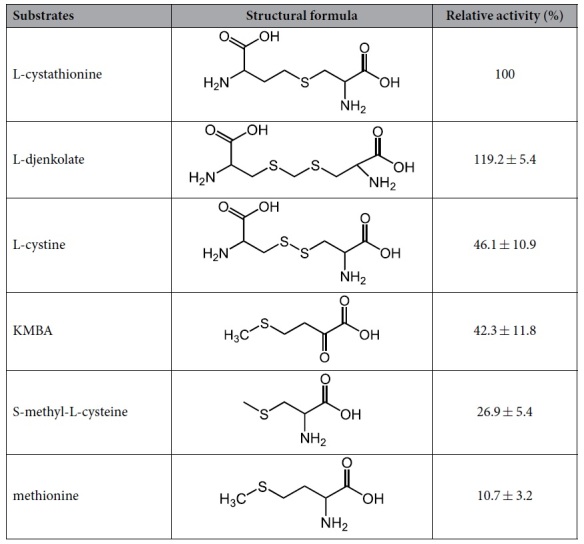
Substrate specificity of purified STR3.

Demethiolation activities with different substrates were determined in the standard enzyme assay system. Specific activity toward L-cystathionine was 22.04 ± 2.40 mM MTL·min^−1^·mg protein^−1^. The relative activity of demethiolation of L-cystathionine to thiol was set at 100%.
